# Multimodal EEG-MRI in the differential diagnosis of Alzheimer's disease and dementia with Lewy bodies

**DOI:** 10.1016/j.jpsychires.2016.03.010

**Published:** 2016-07

**Authors:** Sean J. Colloby, Ruth A. Cromarty, Luis R. Peraza, Kristinn Johnsen, Gísli Jóhannesson, Laura Bonanni, Marco Onofrj, Robert Barber, John T. O'Brien, John-Paul Taylor

**Affiliations:** a Institute of Neuroscience, Newcastle University, Campus for Ageing and Vitality, Newcastle upon Tyne NE4 5PL, UK; b Mentis Cura, Laugavegur 176, 105 Reykjavík, Iceland; c Department of Neurology, Aging Research Center, Ce.S.I., Gabriele d'annunzio, University Foundation, University G. D'annunzio of Chieti-Pescara, Via Fonte Romana, 65124 Pescara, Italy; d Department of Psychiatry, University of Cambridge, Level E4, Box 189, Cambridge CB2 0QC, UK

**Keywords:** MRI, EEG, Alzheimer's disease, Dementia with Lewy bodies, Differential diagnosis, Dopaminergic imaging

## Abstract

Differential diagnosis of Alzheimer's disease (AD) and dementia with Lewy bodies (DLB) remains challenging; currently the best discriminator is striatal dopaminergic imaging. However this modality fails to identify 15–20% of DLB cases and thus other biomarkers may be useful. It is recognised electroencephalography (EEG) slowing and relative medial temporal lobe preservation are supportive features of DLB, although individually they lack diagnostic accuracy. Therefore, we investigated whether combined EEG and MRI indices could assist in the differential diagnosis of AD and DLB.

Seventy two participants (21 Controls, 30 AD, 21 DLB) underwent resting EEG and 3 T MR imaging. Six EEG classifiers previously generated using support vector machine algorithms were applied to the present dataset. MRI index was derived from medial temporal atrophy (MTA) ratings. Logistic regression analysis identified EEG predictors of AD and DLB. A combined EEG-MRI model was then generated to examine whether there was an improvement in classification compared to individual modalities.

For EEG, two classifiers predicted AD and DLB (model: χ^2^ = 22.1, df = 2, p < 0.001, Nagelkerke R^2^ = 0.47, classification = 77% (AD 87%, DLB 62%)). For MRI, MTA also predicted AD and DLB (model: χ^2^ = 6.5, df = 1, p = 0.01, Nagelkerke R^2^ = 0.16, classification = 67% (77% AD, 52% DLB). However, a combined EEG-MRI model showed greater prediction in AD and DLB (model: χ^2^ = 31.1, df = 3, p < 0.001, Nagelkerke R^2^ = 0.62, classification = 90% (93% AD, 86% DLB)).

While suggestive and requiring validation, diagnostic performance could be improved by combining EEG and MRI, and may represent an alternative to dopaminergic imaging.

## 1 Introduction

Dementia with Lewy bodies (DLB) is the second most common form of neurodegenerative dementia following Alzheimer's disease (AD). However, distinguishing DLB from AD continues to be difficult because of common and overlapping clinical and neuropathological features (Galasko, 2001, McKeith et al., 1994), and as such, methods which can improve their diagnostic accuracy and in turn, their management, are of great importance.

Functional imaging approaches such as brain perfusion SPECT studies have showed variable DLB sensitivity (64–85%) and AD specificity (64–87%) (Colloby et al., 2008, Hanyu et al., 2006a, Hanyu et al., 2006b, Lobotesis et al., 2001, Pasquier et al., 2002, Shimizu et al., 2005), while glucose metabolism ^18^F-FDG PET have similarly reported varying DLB sensitivity (64–92%) and AD specificity (65–92%) (Gilman et al., 2005, Ishii et al., 1998, Minoshima et al., 2001). The myocardial scintigraphy SPECT tracer ^123^I-MIBG that detects early disturbances of the sympathetic nervous system has now also emerged as a promising diagnostic marker (e.g. DLB sensitivity 69–100%, AD specificity 89%–100%) (Hanyu et al., 2006a, Inui et al., 2014, Shimizu et al., 2016, Treglia and Cason, 2012, Yoshita et al., 2015) but needs further validation outside specialist centres. Presently, dopaminergic ^123^I-FP-CIT SPECT is, perhaps, the most established diagnostic tool in the differential diagnosis of DLB and AD, where an autopsy study of confirmed cases reported DLB sensitivity 88% and non-DLB specificity 100% (Walker et al., 2007). However FP-CIT still misses DLB cases where there is relatively less nigrostriatal degeneration despite significant cortical Lewy body disease. Furthermore it is not available across all dementia centres and is relatively expensive.

Using magnetic resonance imaging (MRI), patterns of grey matter (GM) atrophy in AD occur predominantly in the medial temporal lobe and temporoparietal association cortices (Burton et al., 2002, Karas et al., 2003, Takahashi et al., 2010, Watson et al., 2012, Whitwell et al., 2007), and the importance of medial temporal atrophy (MTA) is reflected in its inclusion in the revised diagnostic criteria for AD (Dubois et al., 2007, McKhann et al., 2011). In DLB, while there is some degree of overlap with AD in terms of atrophy, changes are often less diffuse and MTA is relatively preserved (Burton et al., 2002, Karas et al., 2003, Takahashi et al., 2010, Whitwell et al., 2007). Relative preservation of MTA has now become a supportive feature of the revised consensus criteria for DLB (McKeith et al., 2005). Assessment of MTA is commonly undertaken by visual rating of MRI scans using the Scheltens scale, which has emerged as a robust, quick, and clinically applicable method of discriminating AD from normal aging and other causes of dementia (Burton et al., 2009, Scheltens et al., 1992), that also correlates with volumetry and AD pathology (Burton et al., 2009). However, normal MTA scores do not exclude a diagnosis of AD, while MTA can also occur in other dementias (Barber et al., 1999, Bastos-Leite et al., 2007).

Electroencephalography (EEG) can also provide another means by which to measure the wide-scale cortical disturbances that occur in dementia and has the advantage of being non-invasive, inexpensive, and relatively simple to use. It is recognised that EEG slowing is among the supportive features for the diagnosis of DLB (McKeith et al., 2005). Whilst it has been estimated that the diagnostic accuracy of spectral and visual EEG analysis is approximately 80%, with good sensitivity, specificity remains poor (Brenner et al., 1988) and can be influenced by other confounders (e.g. medication, physical state etc.). Nevertheless with the advent of semi-automated or fully automated statistical quantitative EEG methods, which consider a range of EEG temporal and spatial features, better delineation of DLB from AD appears possible (Bonanni et al., 2008). However no studies have examined the combination of multimodal approaches, particularly structural imaging and EEG in the diagnosis of DLB. Such approaches may be advantageous in settings where FP-CIT is not available or as an adjunct to further clarifying the diagnosis of DLB in those with a negative FP-CIT result.

While EEG slowing and relative preservation of the medial temporal lobe are supportive features of DLB, individually however they tend to lack diagnostic precision. Therefore, our objective was to investigate the diagnostic utility of implementing combined EEG and visual MRI indices in the differential diagnosis of AD and DLB. Our hypothesis was that greater diagnostic accuracy between AD and DLB would be achieved using a combination marker (EEG-MRI) compared to markers from individual EEG and MRI modalities.

## 2 Materials and methods

### 2.1 Subjects

Fifty one individuals over the age of 60 (30 subjects with probable AD (McKhann et al., 1984), 21 with probable DLB (McKeith et al., 2005)) were recruited from a community dwelling population of patients referred to local Old Age Psychiatry, Geriatric Medicine or Neurology Services. All subjects underwent clinical and neuropsychological assessments. Twenty one similar aged healthy controls were also recruited from among relatives and friends of patients with dementia. The research was approved by the local ethics committee. All subjects or, where appropriate, their nearest relative, provided written informed consent. Exclusion criteria for all subjects included contra-indications for MR imaging, previous history of alcohol or substance misuse, significant neurological or psychiatric history, focal brain lesions on brain imaging or the presence of other severe or unstable medical illness.

Assessments included the Cambridge Cognitive Examination (CAMCOG), incorporating the Mini-Mental State Examination (MMSE) (Folstein et al., 1975), Neuropsychiatric Inventory (NPI) (Cummings et al., 1994) and the clinician's assessment of fluctuation (Walker et al., 2000). Motor parkinsonism was measured with the Unified Parkinson's Disease Rating Scale Part III (UPDRS-III) (Fahn, Elton and Committee, 1987).

### 2.2 MRI

All participants underwent whole brain T1 weighted MR scanning (3D MPRAGE, sagittal acquisition, matrix 216 × 208 × 180, repetition time (TR) = 8.3 m, echo time (TE) = 4.6 m, inversion time (TI) = 1250 m, flip angle = 8°, SENSE factor = 2, voxel output 1 × 1 × 1 mm^3^) on a 3 T MRI system using an 8 channel head coil (Intera Achieva scanner, Philips Medical Systems, Eindhoven, Netherlands). The acquired volumes were angulated such that the axial slice orientation was standardised to align with the anterior commissure-posterior commissure (AC-PC) line.

### 2.3 MRI visual rating

**Fig. 1:**
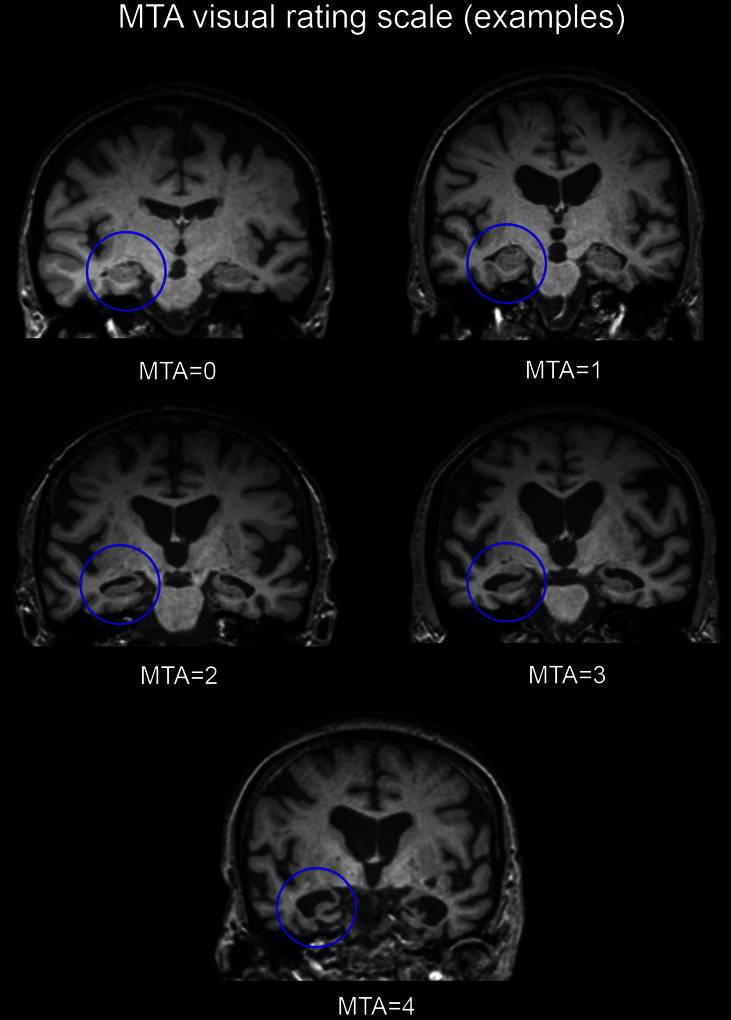
Coronal T1-weighted images depicting bilateral atrophy at each level of the MTA five-point scale.

Scans were assessed by an experienced rater (RB), who was blind to diagnoses and all clinical information. MTA was assessed using Schelten's scale, from coronal sections of T1-weighted images where scores for left and right hemispheres were recorded. The scale rates medial temporal atrophy using a 5 category system according to combination measures of the widths of the choroid fissure and temporal horn as well as the height of the hippocampal formation (0 = normal, 1 = minimal, 2 = mild, 3 = moderate, 4 = severe). Fig. 1 illustrates specific examples of bilateral MTA for each category. Left and right scores were summed to give an overall combined MTA score (maximum 8). Three subjects were then randomly chosen by an independent observer and repeat measurements were taken over five consecutive days by the same rater (RB) to determine intra-observer reliability.

### 2.4 EEG

**Fig. 2:**
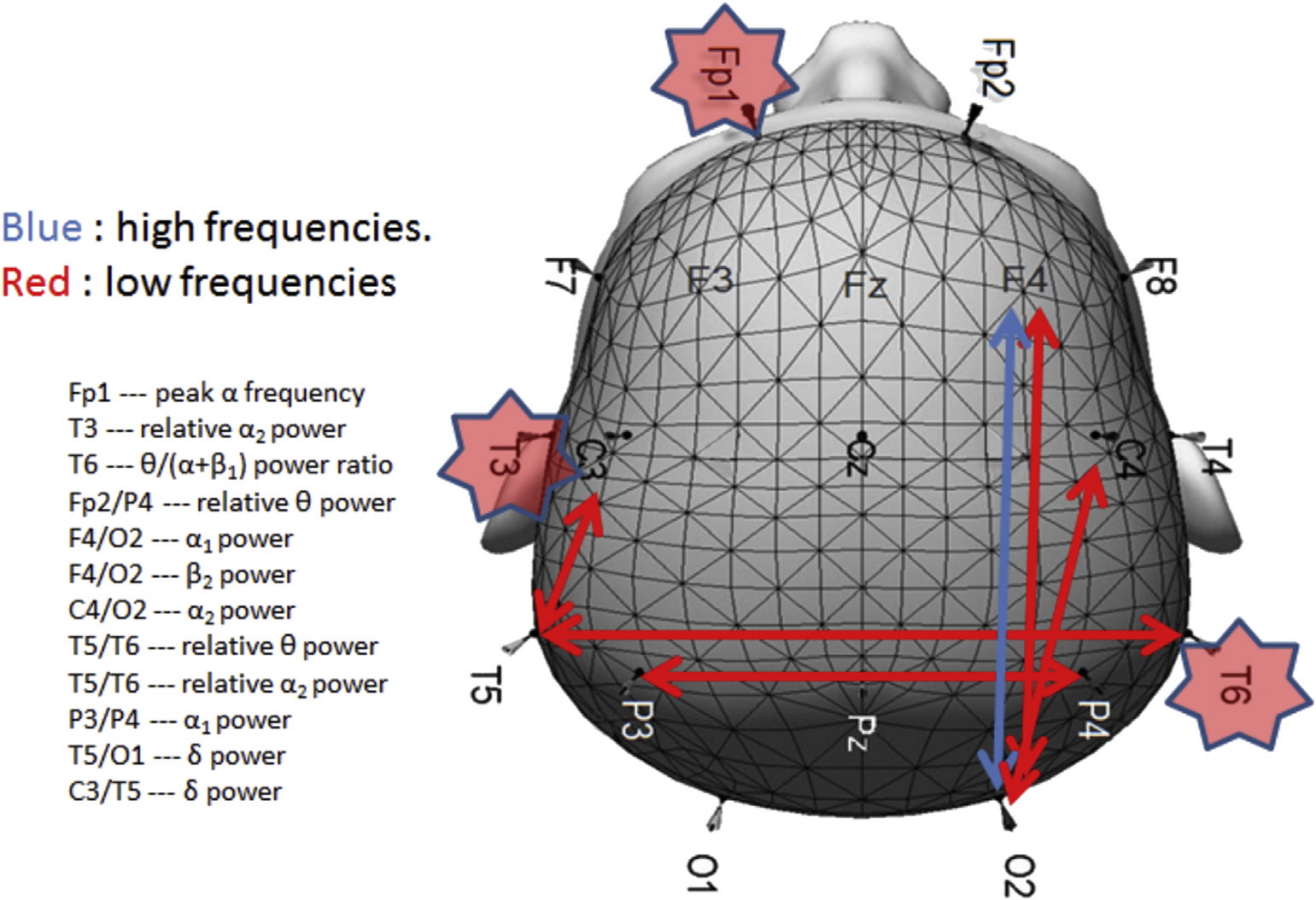
Schematic of electrode locations and features that constructed the ADms-LP classifier. Arrows indicate coherences between electrodes, while stars depict single electrode qEEG parameters.

All participants underwent high density EEG resting-state recordings for a duration of 2 min and 30 s. Participants were seated throughout the recordings and instructed to remain as still as possible. EEG data were acquired using Wave guard caps (ANT Neuro, Netherlands) comprising 128 sintered Ag/AgCl electrodes placed according to the 10–5 positioning system (Oostenveld and Praamstra, 2001). Channel signals were recorded using ASA-Lab software (ANT Neuro, Netherlands) with a sampling frequency of 1024 Hz and electrode impedances of <5 kΩ. All electrodes were referenced to Fz and a ground electrode was attached to the clavicle. Continuous EEG data files were saved and stored for off-line processing. For the purposes of EEG analysis only 19 electrodes (on the basis of the 10–20 system) were utilised: Fp1, Fp2, F3, F4, F7, F8, Fz, T3, T4, T5, T6, C3, C4, Cz, P3, P4, Pz, O1, and O2 (Fig. 2).

### 2.5 EEG analysis

A number of EEG classifiers that differentiated specific pairs of subject groups were derived from an independent Nordic based dataset which examined 654 participants (226 healthy controls, 239 AD, 52 DLB, 147 other diagnoses), recruited from the Memory Clinic of the Geriatric Department, National University Hospital, Reykjavik, Iceland (Snaedal et al., 2012). In brief, classifiers were generated from pairings of groups (e.g. LBD vs. AD, Controls vs. AD, and Controls vs. LBD) in the Nordic cohort using statistical pattern recognition (SPR). Twenty spectral features were identified as well as 37 associated coherence features leading to a total of 1120 feature extractions from each EEG recording. Classifiers, based on 20 of the feature extractions were obtained by comparing two different subject groups A and B, and indexed with a score between 0 and 1; where a value close to 0 was indistinguishable from the EEG's in group A, and a value closer to 1 was indistinguishable from the EEGs in group B. A genetic algorithm (Engedal et al., 2015), was then applied to select features used in the construction of the classifier for each pair of groups. The target value of the genetic evolution of classifiers was the area under the receiver operating characteristic (ROC) curve and optimisation based upon achieving a good-to-excellent sensitivity and specificity suitable for clinical utility. A 10-fold cross-validation approach was then used to obtain average values for accuracy, sensitivity and specificity for each classifier, and standard deviations were estimated from bootstrap resampling. Six classifiers (Table 1) identified from this study were then applied to our EEG dataset, and classifier scores were calculated for all participants. Fig. 2 shows example EEG features that were used to construct the moderate-severe AD vs. dementia with Lewy bodies or Parkinson's disease dementia (ADms-LP) classifier.

### 2.6 Statistical analyses

Data were exported into the Statistical Package for Social Sciences software (SPSS ver. 22.0, http://www-01.ibm.com/software/analytics/spss/products/statistics/) for further statistical evaluation. Continuous variables were tested for normality of distribution using the Shapiro-Wilk test and visual inspection of histograms. Differences in demographic, clinical and imaging variables were examined where appropriate using parametric (ANOVA) and non-parametric (χ^2^, Kruskal-Wallis, Mann-Whitney U) tests. Logistic regression analyses (LRA) were conducted to investigate EEG and MRI predictors of controls, AD and DLB. More specifically, forward stepwise approaches were used to identify the most significant EEG classifiers that predicted the groups. This involved successively adding and then removing the EEG classifiers in accordance with pre-existing statistical criteria of their parameter estimates. For MRI and subsequent combined EEG-MRI logistic regression models, the ‘enter’ method was applied thereby including and retaining all variables. Assuming clinical diagnosis as the ‘gold standard’, diagnostic characteristics of the models in distinguishing groups were determined from ROC curves. To quantify intra-rater reliability of MTA scores, a two-way mixed single measure intra-class correlation coefficient (ICC) was evaluated. A p-value of ≤0.05 was considered significant.

## 3 Results

### 3.1 Subject characteristics

Table 2 shows demographic and group characteristics. Groups were matched for age and gender. CAMCOG and MMSE scores were similar between AD and DLB but differed from controls. As expected, UPDRS III measures were significantly higher in DLB than AD and controls. NPI, NPI_hallucinations and CAF scores were all significantly greater in DLB than AD. The proportion of individuals receiving cholinesterase inhibitors did not significantly differ between dementia groups. In diagnosing DLB, 12 patients (57%) had all 3 core symptoms of Parkinsonism, visual hallucinations and cognitive fluctuations, whereas 5 patients (24%) had 2 core symptoms while the remaining 4 patients (19%) had 1 core symptom but these individuals all had a history of REM sleep behaviour disorder (RBD). RBD was present in 16 patients with DLB (76%). Of all DLB subjects studied, 12 had dopamine transporter ^123^I-FP-CIT imaging (11 positive, 1 negative).

### 3.2 EEG classifier scores and MTA ratings

EEG classifier scores which represent the probability of a subject belonging to a particular category within a classifier are summarised for controls, AD and DLB (Table 3). For MTA ratings, frequency of summed scores in controls, AD and DLB are presented in Table 4. Significantly higher MTA scores were observed in AD compared to DLB and controls (p < 0.03), where the highest proportion of AD's (40%) were rated as having ‘moderate’ MTA, while for most DLB's (52%) and controls (90%), MTA was rated as ‘minimal’. Intra-observer reliability was also found to be ‘excellent’ (ICC: 0.95 for both left and right scores).

### 3.3 EEG and MRI models

#### 3.3.1 Controls vs. AD

For EEG, ‘forward stepwise’ LRA showed that ADms_CLR (β = 5.2, e^β^ = 172.8 (95% CI: 8.8–3375.4), Wald χ^2^ = 11.5, p = 0.001) significantly predicted controls and AD (model: χ^2^ = 16.6, df = 1, p < 0.001, Nagelkerke R^2^ = 0.38, classification accuracy = 77% (Con 62%, AD 87%)). In a separate model, MTA (β = 1.5, e^β^ = 4.3 (95% CI: 1.8–10.1), Wald χ^2^ = 10.7, p = 0.001) also significantly predicted controls and AD (model: χ^2^ = 34.0, df = 1, p < 0.001, Nagelkerke R^2^ = 0.66, classification accuracy = 82% (Con 91%, AD 77%)). However, a combined EEG-MRI model of ADms_CLR (β = 2.8, e^β^ = 15.8 (95% CI: 0.5–525.9), Wald χ^2^ = 2.4, p = 0.1) and MTA (β = 1.3, e^β^ = 3.6 (95% CI: 1.5–8.8), Wald χ^2^ = 8.0, p = 0.005) was then derived and showed superior classification (model: χ^2^ = 36.6, df = 2, p < 0.001, Nagelkerke R^2^ = 0.69, classification accuracy = 90% (Con 86%, AD 93%)).

#### 3.3.2 AD vs. DLB

For EEG, ‘forward stepwise’ LRA revealed ADms_CLR (β = 5.0, e^β^ = 151.4 (95% CI: 1.5–15784.6), Wald χ^2^ = 4.5, p = 0.03) and ADms_LP (β = 7.5, e^β^ = 1720.6 (95% CI: 15.9–186496.7), Wald χ^2^ = 9.7, p = 0.002) significantly predicted AD and DLB (model: χ^2^ = 22.1, df = 2, p < 0.001, Nagelkerke R^2^ = 0.47, classification accuracy = 77% (87% AD, 62% DLB)). In a separate model, MTA was also found to be a significant predictor (β = −0.40, e^β^ = 0.7 (95% CI: 0.5–0.9), Wald χ^2^ = 5.5, p = 0.02) of AD and DLB (model: χ^2^ = 6.5, df = 1, p = 0.01, Nagelkerke R^2^ = 0.16, classification accuracy = 67% (77% AD, 52% DLB). However, a combined EEG-MRI model of ADms_CLR (β = 6.2, e^β^ = 508.8 (95% CI: 1.4–185885.4), Wald χ^2^ = 4.3, p = 0.04), ADms_LP (β = 10.2, e^β^ = 27732.5 (95% CI: 20.9–36711282.9), Wald χ^2^ = 7.8, p = 0.005) and MTA (β = −0.64, e^β^ = 0.5 (95% CI: 0.3–0.9), Wald χ^2^ = 6.3, p = 0.01) was then generated that showed greater accuracy in categorising AD and DLB (model: χ^2^ = 31.1, df = 3, p < 0.001, Nagelkerke R^2^ = 0.62, classification accuracy = 90% (93% AD, 86% DLB)).

#### 3.3.3 Diagnostic utility

**Fig. 3:**
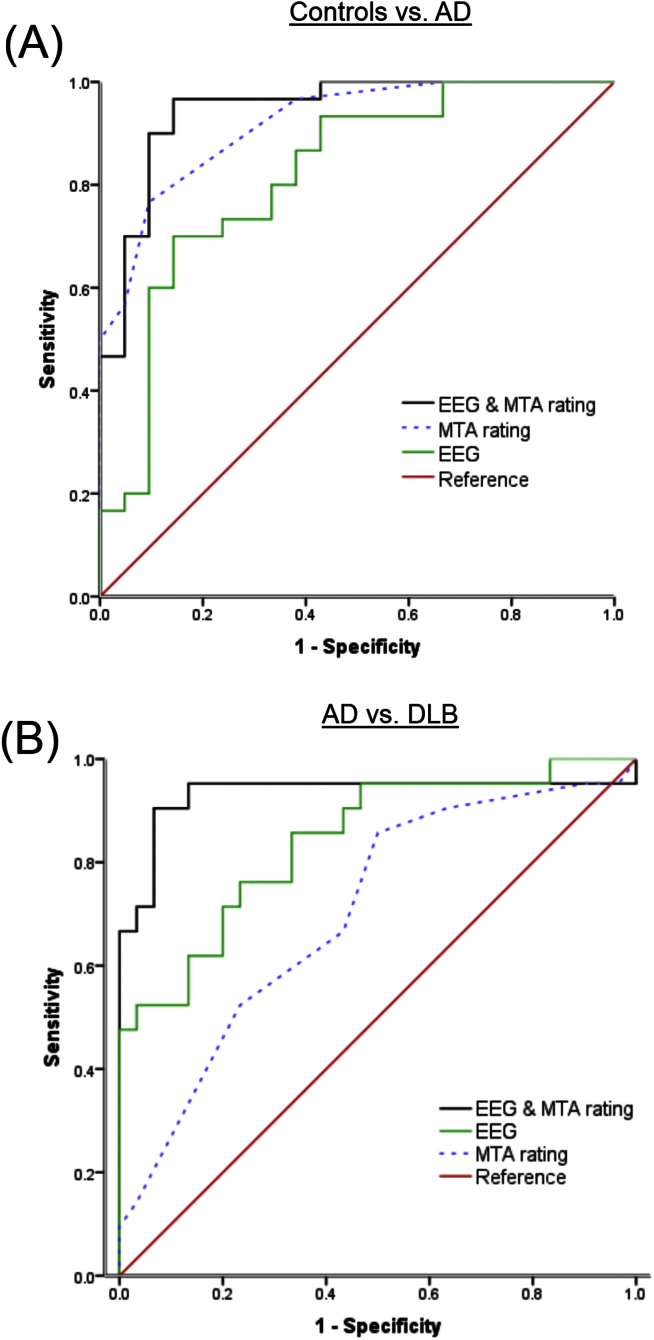
Diagnostic performance of MRI (MTA rating), EEG and combined markers in controls vs. AD and AD vs. DLB using ROC curve analysis.

Diagnostic characteristics of EEG, MRI and EEG-MRI models in controls vs. AD and AD vs. DLB are summarised in Table 5. In distinguishing controls from AD, diagnostic performance was ‘excellent’ for MTA rating (ROC area 0.92), ‘good’ for EEG (ROC area 0.82) and ‘excellent’ for the combined EEG-MRI model (ROC area 0.95). Fig. 3A shows the ROC curves. Optimal (minimum false positive and false negative rates) corresponded to AD sensitivity 90% and control specificity 91% for the EEG-MRI case. In distinguishing DLB from AD, diagnostic accuracy was ‘moderate’ for MTA rating (ROC area 0.70), ‘good’ for EEG (ROC area 0.84) and ‘excellent’ for the combined EEG-MRI model (ROC area 0.93). Fig. 3B depicts the ROC curves. Optimal rates corresponded to DLB sensitivity 91% and AD specificity 93% for the EEG-MRI model.

## 4 Discussion

To our knowledge this is the first study to investigate the implementation of combined EEG biomarkers and MRI visual rating scores in the differential diagnosis of DLB and AD. Our major finding was that in controls vs. AD and for the clinically relevant DLB vs. AD, a joint EEG-MRI model demonstrated greater classification (90%) and diagnostic accuracy compared to individual modalities.

A number of studies have now started to investigate the diagnostic utility of EEG in the differential diagnosis of DLB and AD. Using structured visual rating scales, patients with DLB could be distinguished from those with AD with sensitivity 72–79% and specificity 76–85% (Lee et al., 2015, Roks et al., 2008). Bonanni and colleagues also showed the potential of EEG in differentiating AD from DLB by application of advanced statistical methods to quantitative EEG measures (Bonanni et al., 2008), while others also reported that EEG parameters could be used to distinguish DLB from AD with ROC curve areas between 0.75 and 0.80 (Andersson et al., 2008). Utilising statistical pattern recognition algorithms to generate DLB/Parkinson's disease classifiers as discriminatory variables, Snaedal J et al. and Engedal K et al. described excellent diagnostic characteristics among DLB and AD (ROC area 0.97, DLB sensitivity 93%, AD specificity 86%) and (0.92, 85%, 87%) respectively (Engedal et al., 2015, Snaedal et al., 2012). The forward projection of the classifiers derived from Engedal et al. cohort onto our dataset found that diagnostic measures with EEG were ‘good’ (0.84, 76%, 77%), thus further validating the use of classifiers as a potential diagnostic method for differentiating DLB from AD.

For MTA ratings on MRI, significantly higher MTA scores were observed in AD compared to DLB and controls. The MTA profile (AD > DLB > Controls) is consistent with our previous and independent visual rating dementia study, where MTA scores were also found to be significantly higher in AD and DLB compared to controls as well as greater in AD than DLB (O'Donovan et al., 2013). Using MTA scores as the discriminatory variable, diagnostic accuracy was ‘moderate’ (0.70, 67%, 57%) in differentiating DLB from AD, suggesting a supportive rather than an absolute diagnostic marker.

Combining the biomarkers of EEG and MRI resulted in enhanced diagnostic accuracy relative to each individual modality. Of note, for the DLB vs. AD contrast, of the 10 DLB participants incorrectly categorised as AD by MRI, 8 were correctly classified as DLB by EEG; while of the 8 DLBs incorrectly categorised as AD by EEG, 6 were correctly classified as DLB by MRI. Therefore, this suggests that the combined model appears to be harvesting, in a synergistic manner, different elements of the AD and DLB phenotypes from EEG and MRI, enabling relatively high diagnostic classification. Examination of our data suggested that MRI had a tendency to categorise AD and DLB with high and low MTA respectively and with a longer duration of illness. In contrast, EEG largely appeared to group AD patients with shorter duration of illness, less cognitive impairment and relatively less MTA as well as, contrastingly, those DLB patients with higher MTA and greater deficits in global cognition. Although requiring replication in an independent cohort, a pattern emerges which suggests that EEG largely classify less disease progressed patients while MRI categorises more established cases, hence, these different modalities may be effective at different disease stages thus allowing enhanced differential diagnostic utility when comparing DLB and AD. However given misclassified numbers were relatively small, and an absence of longitudinal data, our assertions can only be speculative.

Strengths of the present study are: multimodal imaging and rigorous clinical and neuropsychological data of subjects. Furthermore, EEG classifiers were derived from an independent cohort enhancing their robustness as potential biomarkers. Further details of how to obtain and implement these EEG classifiers can be found using the following link (http://www.mentiscura.com/sigla/). Weaknesses were the lack of autopsy confirmed diagnoses and relatively small patient numbers.

In conclusion, although suggestive, it appears that the diagnostic accuracy between DLB and AD could be enhanced by combining EEG and MRI parameters and thus may represent alternative or adjunctive biomarkers to dopaminergic imaging. Further studies are warranted with large autopsy confirmed populations in order to reveal its true diagnostic extent. Whilst FP-CIT SPECT remains the best validated imaging biomarker for DLB to date, and so the “gold standard”, a simple EEG-MRI marker shows promise, and, if replicated by others, may prove a useful alternative to not only dopaminergic but to radionuclide imaging.

## Author contributions

Sean J. Colloby co-designed the study, carried out the image and data analyses and co-wrote the manuscript.

Ruth A. Cromarty collected both EEG and neuropsychological data and reviewed the manuscript.

Luis R. Peraza reviewed the manuscript.

Kristinn Johnsen generated the EEG classifiers and contributed to manuscript writing.

Gísli Jóhannesson reviewed the manuscript.

Laura Bonanni reviewed the manuscript.

Marco Onofrj reviewed the manuscript.

Robert Barber conducted the MRI visual ratings and reviewed the manuscript.

John T. O'Brien reviewed the manuscript.

John-Paul. Taylor co-designed the study, obtained funding and co-wrote the manuscript.

All authors have seen and agree with the contents of the manuscript and guarantee the accuracy of the references.

## Role of funding source

The work was supported by: the Newcastle Healthcare Charity (BH0070250); Academy of Medical Sciences, Wellcome Trust Starter Grants scheme for Clinical Lecturers (BH090112 to J-P.T.); Wellcome Intermediate Clinical Fellowship (BH083281 to J-P.T.); National Institute for Health Research (NIHR) Newcastle Biomedical Research Centre in Ageing and Chronic Disease and Biomedical Research Unit in Lewy Body Dementia based at Newcastle upon Tyne Hospitals NHS Foundation Trust and Newcastle University; NIHR Dementia Biomedical Research Unit at Cambridge University Hospitals NHS Foundation Trust and the University of Cambridge. The views expressed are those of the author(s) and not necessarily those of the NHS, the NIHR or the Department of Health.

## Conflicts of interest

The material is original research, has not been previously published and has not been submitted for publication elsewhere. Dr's Colloby, Cromarty, Peraza, Johnsen, Jóhannesson, Bonanni, Onofrj, Barber declare no competing financial interests. Dr Taylor has been a consultant of Lundbeck, received honoraria for talks from GE Healthcare and Flynn pharmaceuticals as well as travel expenses from Mentis Cura. Professor O'Brien has been a consultant for GE Healthcare, Lilly, Bayer Healthcare, TauRx and Nutricia and has received honoraria for talks from GE Healthcare, Lilly and Novartis.

## Figures and Tables

**Table 1:** The composition of groups in EEG based A vs. B classifiers.

Classifier	Group A	Group B
NRM_CL	NRM	sMCI, pAD, AD, ADms, LP, VaD, FTD, DPR
sMCI_CLR	sMCI	pAD, AD, ADms, LP, VaD, FTD, DPR
AD_CLR	pAD, AD	sMCI, ADms, LP, VaD, FTD, DPR
ADms_CLR	ADms	sMCI, pAD, AD, LP, VaD, FTD, DPR
LP_CLR	LP	sMCI, pAD, AD, ADms, VaD, FTD, DPR
ADms_LP	ADms	LP

NRM: control; sMCI: stable mild cognitive impairment; pAD: prodromal AD; AD: mild AD; ADms: moderate or severe AD; LP: dementia with Lewy bodies or Parkinson's disease dementia; VaD: vascular dementia; FTD: frontotemporal dementia; DPR: depression; CL: all clinical groups; CLR: corresponding complement of clinical groups.

*Detailed description of classifiers and group definitions are reported in Engedal et al., (Engedal et al., 2015).

**Table 2:** Demographic and group characteristics.

	Controls	AD	DLB	Statistic, p value
*n*	21	30	21	
Gender (m: f)	14: 7	22: 8	15: 6	χ^2^ = 0.3, 0.9
Age (yrs.)	76.2 ± 5.3	77.4 ± 7.8	76.7 ± 6.2	F_2,69_ = 0.2, 0.8
MMSE	77.4 ± 7.8	20.8 ± 3.9	22.4 ± 4.6	**F**_**2,69**_ = **36.2, <0.001**^a^
CAMCOG	77.4 ± 7.8	68.5 ± 13.6	72.3 ± 15.4	**F**_**2,69**_ = **36.5, <0.001**^a^

NPI_total	Na	6.5 ± 6.2	10.0 ± 6.8	**U**_**49**_ = **433.5, 0.02**
NPI_hall	Na	0.03 ± 0.2	1.7 ± 2.0	**U**_**49**_ = **504.0, <0.001**
UPDRS III	1.1 ± 1.4	2.5 ± 2.2	16.8 ± 8.0	**H**_**2**_ = **47.6, <0.001**^b^
CAF	Na	0.6 ± 1.4	4.2 ± 4.2	**U**_**49**_ = **482.5, <0.001**

ChI use (y: n)	Na	28: 2	19: 2	χ^2^ = 0.1, 0.7
DaTSCAN (y: n)	Na	Na	12: 9	

Values expressed as Mean ± 1 SD.

MMSE = Mini mental state examination, CAMCOG = Cambridge cognitive examination, NPI_total = Total neuropsychiatric inventory score, NPI_hall = NPI hallucinations, UPDRS III = Unified Parkinson's disease rating scale (Section III), ChI = Cholinesterase inhibitor, CAF = Clinical assessment of fluctuation, Na = Not applicable.

**Bold** text denotes statistical significance.

Post Hoc tests:

a Con > AD, DLB (p < 0.001), AD vs. DLB (p ≥ 0.3) (Gabriel's).

b DLB > Con, AD (p < 0.001), Con vs. AD (p = 0.2) (Mann-Whitney U).

**Table 3:** Mean scores (probabilities) of Controls, AD and DLB belonging to a particular classifier.

Classifier	Controls (n = 21)	AD (n = 30)	DLB (n = 21)
NRM_CL	0.53 ± 0.20	0.42 ± 0.22	0.23 ± 0.24
sMCI_CLR	0.64 ± 0.15	0.50 ± 0.19	0.48 ± 0.15
AD_CLR	0.54 ± 0.18	0.43 ± 0.17	0.36 ± 0.15
ADms_CLR	0.33 ± 0.25	0.63 ± 0.22	0.72 ± 0.20
LP_CLR	0.27 ± 0.16	0.58 ± 0.19	0.74 ± 0.21
ADms_LP	0.24 ± 0.28	0.10 ± 0.09	0.37 ± 0.33

Values expressed as Mean ± 1 SD.

**Table 4:** Frequency of summed MTA scores across groups.

	Controls	AD	DLB	Statistic, p value
*n*	21	30	21	
MTA	1.2 ± 1.1	4.3 ± 1.9	2.9 ± 1.9	**H**_**2**_ = **29.2, <0.001**^a^
0–2	19 (90%)	7 (23%)	11 (52%)	
3–4	2 (10%)	8 (27%)	7 (33%)	
5–6	0 (0%)	12 (40%)	2 (10%)	
7–8	0 (0%)	3 (10%)	1 (5%)	

Values expressed as Mean ± 1 SD. MTA = Medial temporal atrophy.

Values in parentheses indicate frequency expressed as percentage.

**Bold** text denotes statistical significance.

Post Hoc tests:

a AD > Con (p < 0.001), DLB > Con (p = 0.003), AD > DLB (p = 0.03) (Mann-Whitney U).

**Table 5:** Diagnostic performance of EEG, MRI and combined EEG-MRI markers.

	ROC curve area (Area ±SE)	Sensitivity^a^ (%)	Specificity^a^ (%)
*Controls vs. AD*			
MRI	0.92 ± 0.04	77	91
EEG	0.82 ± 0.06	70	86
EEG-MRI	0.95 ± 0.03	90	91
*AD vs. DLB*			
MRI	0.70 ± 0.08	67	57
EEG	0.84 ± 0.06	76	77
EEG-MRI	0.93 ± 0.05	91	93

SE = Standard Error.

Values in parentheses indicate occurrence expressed as percentage.

a Minimum false positive and false negative results (Maximum Youden's J statistic).
